# Lip Dose Challenges in Food Allergy: Current Practice and Diagnostic Utility in the United Kingdom

**DOI:** 10.1016/j.jaip.2019.04.037

**Published:** 2019

**Authors:** Marta Vazquez-Ortiz, Siân Ludman, Antony Aston, Lee Noimark, Paul J. Turner

**Affiliations:** a Section of Paediatrics (Allergy and Infectious Diseases) & MRC and Asthma UK Centre in Allergic Mechanisms of Asthma, Imperial College London, London, United Kingdom; b Department of Paediatric Allergy, Imperial College Healthcare NHS Trust, London, United Kingdom; c Department of Paediatric Allergy, Royal Devon and Exeter NHS Foundation Trust, Exeter, United Kingdom; d Department of Paediatrics, Barts Health NHS Trust, London, United Kingdom; e University of Sydney, Sydney, New South Wales, Australia

**Keywords:** Food allergy, Children, Diagnosis, Oral food challenge, Lip dose, Labial challenge, LDC, Lip dose challenge, OFC, Oral food challenge

## Abstract

**Background:**

Lip dose challenges (LDCs) are often performed as an initial step before oral food challenges (OFCs). However, guidance on how to perform and interpret LDCs is unclear, and data are lacking regarding the diagnostic accuracy of LDCs.

**Objective:**

To investigate current practice with respect to LDCs among UK allergy health care professionals, and to evaluate the diagnostic utility of LDCs in children undergoing OFCs for IgE-mediated food allergy.

**Methods:**

We used an electronic survey to assess the use of LDCs by UK Allergy clinics. Separately, we prospectively recruited children undergoing “low-risk” OFCs for suspected IgE-mediated food allergy from 2 large specialist allergy units in London. LDC was performed 30 minutes before the OFC, by applying the food to the inner lip for 30 seconds. Objective symptoms were considered a positive outcome. All patients subsequently proceeded to OFC regardless of LDC outcome, and outcome assessed according to PRACTALL consensus.

**Results:**

We received 147 responses to the online survey, representing 67% of registered pediatric allergy clinics in the United Kingdom. Eighty percent of respondents (representing 81% of responding centers) included LDC as the first step of OFC in routine clinical practice. There was a wide variation in both how LDCs were performed and interpreted, with one-third not proceeding to OFC if LDC resulted in subjective symptoms. In the prospective study, 198 children (mean age, 7 years) with conclusive OFCs were included. Foods tested were tree nuts (30%), peanut (16.6%), egg (16%), fish (10.5%), milk (6%), shrimp (4%), and other (16.9%). There were 12 positive LDCs (1 of which triggered systemic symptoms: generalized urticaria) and 31 positive OFCs. Two children with positive LDCs went on to have a negative diagnostic OFC. Sensitivity of LDC was 32%, specificity 98%, with a false-negative rate of 68%.

**Conclusions:**

Most UK allergy clinics included LDC as an initial step during OFC, despite a wide variation in how LDCs are performed and interpreted, which raises major concerns about the reproducibility and the validity of the test. We found that LDC had poor sensitivity as an alternative or initial step to formal OFC.

***What is already known about this topic?*** Lip dose challenges (LDCs) may be common in clinical practice.

***What does this article add to our knowledge?*** Most UK allergy centers include a lip dose as part of routine food challenge, with a wide variation in performance and interpretation, which impacts on test reproducibility and validity. The diagnostic utility of LDCs is limited.

***How does this study impact current management guidelines?*** LDC is unlikely to confer any advantage if incorporated into routine oral food challenges, and may be associated with false positives and systemic reactions. Clinical guidelines should actively caution against the use of LDC in the diagnosis of food allergy.

## Introduction

Oral food challenges (OFCs) remain the criterion standard for the diagnosis of IgE-mediated food allergy.^1^, ^2^ Anecdotally, lip dose challenges (LDCs) (also referred to as labial challenges in the literature) appear to be commonplace in clinical practice in some countries, although guidance on how to perform and interpret LDCs is lacking. Monteret-Vautrin et al^3^ suggested an application time between 2 seconds and 10 minutes, a wide range that is likely to have an impact on outcome. Likewise, very subtle changes such as “smoothing of the lip” could be considered sufficient to indicate a positive LDC,^3^ which may lead to subjectivity in interpretation. There is a lack of data as to how the LDC correlates to the OFC outcome: previous studies assumed a diagnosis of food allergy on the basis of a positive LDC, without progressing to formal OFC to confirm the diagnosis.^4^, ^5^ Hourihane et al^6^ reported that a subset of patients with suspected peanut allergy and a positive LDC passed a subsequent OFC, raising a concern about the risk of false-positive LDCs. Accordingly, LDCs are not recommended in some guidelines on food allergy diagnosis or OFCs,^2^, ^7^ but are included as a first step in other protocols.^8^, ^9^

Given this variation in both practice and interpretation, we undertook a 2-phase study: first, to systematically assess the use of LDC, and investigate current practice and perceptions of LDC among health care professionals working in Pediatric Allergy in the United Kingdom; second, to evaluate the diagnostic utility of LDC compared with OFC outcomes in children undergoing diagnostic food challenge for suspected IgE-mediated food allergy.

## Methods

### Survey of health care professionals

We undertook a web-based survey among health care professionals who were members of the Paediatric Allergy Interest Group of the British Society for Allergy and Clinical Immunology. The content of the questionnaire is presented in Table E1 in this article's Online Repository at www.jaci-inpractice.org. Briefly, it covered respondents' demographic characteristics, professional experience, and perception of LDC utility in clinical practice. Also, respondents were asked how they would perform LDC for a fluid, semisolid, and solid food (ie, milk, peanut butter, and cashew, respectively) and how they would interpret the LDC outcome (ie, whether a given manifestation would be considered a positive outcome and whether food allergy diagnosis would be assumed with no need for further OFC).

### Prospective assessment of false positives/negatives associated with LDC

In the second phase of the study, children undergoing open OFC for suspected IgE-mediated food allergy were recruited prospectively from 2 large specialist allergy units in London (St Mary's Hospital [Imperial College Healthcare NHS Trust] and the Royal London Hospital [Barts Health NHS Trust]), from June to December 2015. Clinical data were registered including age, atopic dermatitis, other food allergies, food tested at OFC, skin prick test result to the tested food, and whether the suspected diagnosis was based on previous reaction or sensitization only. Skin prick testing was performed according to standard guidelines, with histamine 10 mg/mL as a positive control.^10^ OFC were performed because of indeterminate allergic status—either a reducing skin prick test in the context of possible resolution, level of sensitization less than 95% positive predictive values, or more than 95% predictive value but with an absence of previous reaction or with evidence of possible tolerance.

LDC was performed in all subjects 30 minutes before OFC. A small pea-sized amount of the allergen in question was applied to the border of the inner and outer lip and the patient observed for 30 minutes; we chose this time interval to be consistent with the dosing interval used for OFC, although existing LDC protocols use a 20-minute interval.^8^, ^9^ We used a liquid/paste (eg, nut butter) for application; where this was not possible due to the nature of the food, the lip was gently rubbed with the allergen in question for 10 seconds. The following objective symptoms following LDC were considered a positive outcome: erythema, urticaria, and/or angioedema at the application site or systemic skin, gastrointestinal, respiratory, or cardiovascular symptoms. Subjective symptoms were not used as indicative of a positive LDC.

All subjects then proceeded to open OFC regardless of the LDC outcome, except where the LDC resulted in a systemic reaction that would qualify as a positive result in an OFC. Children were fed incremental doses of the allergen in question, up to a top dose of 3 g protein equivalent, at 30-minute intervals. The challenge was halted at onset of objective clinical symptoms, in line with PRACTALL criteria,^1^ unless consent was withdrawn previously. Children were observed for a further 2 hours following the last dose. Each OFC was documented on a standardized proforma, and outcome reviewed independently by 2 authors (M.V.O., P.J.T.) according to the PRACTALL consensus.^1^ Children with inconclusive OFC (eg, OFC halted before reaching PRACTALL stopping criteria) were excluded from analysis.

LDC was already included in the existing clinical protocols at both sites; thus, the study involving patients was considered a clinical service evaluation. Our local Research Ethics Committee advised that this did not require ethical approval, because no changes were required in relation to routine practice.

### Data analysis

The OFC outcome was used as the reference parameter to calculate the diagnostic performance of the LDC, together with 95% CIs according to the efficient-score method (corrected for continuity) outlined by Newcombe.^11^ Given our interest in assessing the risk of false positives associated with LDC, we used the method of Carley et al^12^ to determine that a sample size of approximately 220 would be sufficient to allow us to estimate the specificity of LDC within 10% of that for OFC with probability of finding a false-positive result at 5% or less. Fisher Exact test was used for comparative analysis of clinical data between children having positive and negative LDC, except for continuous variables for which Mann-Whitney *U* test was used. PASW Statistics 18 (SPSS Inc, Chicago, Ill) was used for analysis.

## Results

### Perception and use of LDCs by health care professionals in the United Kingdom

One hundred forty-seven respondents completed the questionnaire, which represented 67% (53 of 79) of British Society for Allergy and Clinical Immunology–registered pediatric allergy clinics in the United Kingdom. Of these respondents, 88 (60%) were doctors, 51 (32%) specialist nurses, and 8 (5%) dietitians. The vast majority worked in specialist allergy clinics and district general hospitals (48% and 50%, respectively), with 67% working exclusively in the pediatric setting. Overall responses are presented in Table E1.

**Figure 1:**
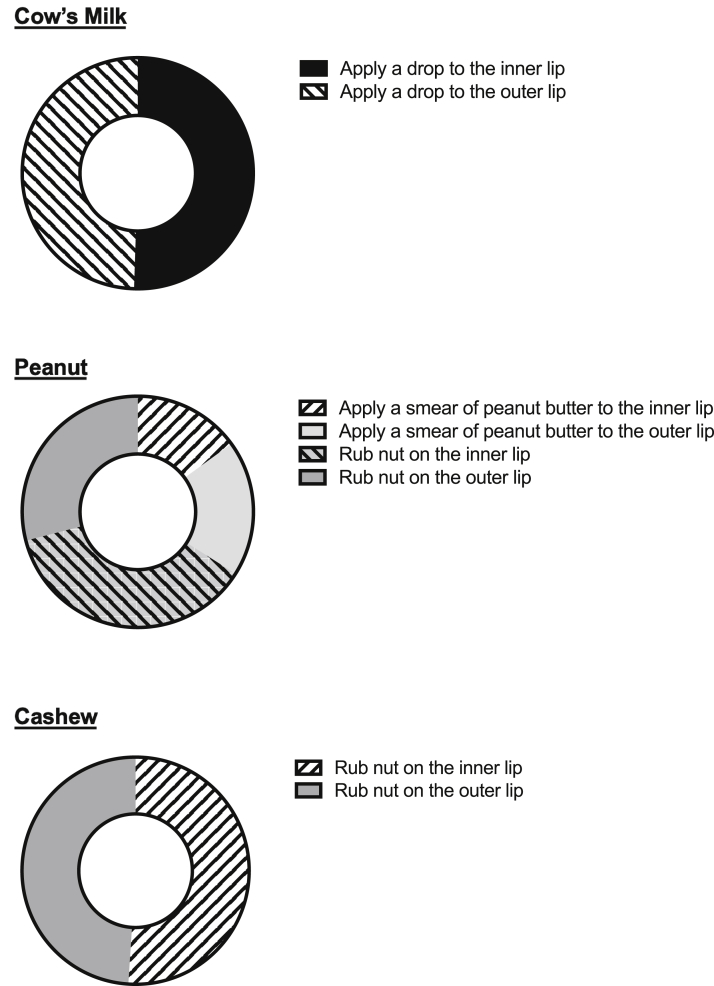
Technique of application for fluid (milk), semisolid (peanut butter), and solid food (cashew) for LDC, as reported by respondents.

Most respondents (71%, 102 of 147) reported that in their opinion, LDCs were a useful first step when performing an OFC. Eighty percent (117 of 147), representing 81% of responding centers, included LDC as a first step in an OFC in routine clinical practice. There was a wide variation in how LDCs were performed, depending on the nature of then food being tested: in particular, there was significant disagreement on whether to apply the food to the inner lip or the outer lip (Figure 1). With respect to the interpretation of LDC, a (subjective) itchy lip or mouth was considered a positive finding by 37% of respondents, erythema at the application site by 46%, and urticaria/angioedema at the application site by 93%. Interestingly, the occurrence of urticaria away from the site of application was considered a positive finding by fewer respondents: 74% if on the face but away from the application site, and 70% if present on the body. There was also significant variation in interpreting outcomes from LDC: 17% and 31% would not proceed to a formal OFC in the presence of oral itch or local erythema, respectively, in contrast to more objective symptoms where most respondents would stop the challenge test and interpret LDC as indicative of food allergy (Table I).

**Table I:** Interpretation of subjective and objective symptoms at LDC

Symptom	STOP the challenge	CONTINUE with oral challenge
Oral pruritus or itchy lip	17%	83%
Erythema at the site of application	31%	69%
Urticaria and/or local angioedema at the site of application	88%	13%
Urticaria/angioedema on face, but remote from the site of application	93%	7%
Urticaria elsewhere (not on face)	93%	7%

### Diagnostic performance of LDC in relation to OFC outcomes

Two hundred twenty children were prospectively recruited for the study, 22 of whom were excluded because of an inconclusive OFC outcome, leaving 198 for analysis. Patients' demographic characteristics are presented in Table II. Twelve children had a positive LDC: 10 with objective skin symptoms at the application site, 1 with facial urticaria, and another who developed generalized urticaria. The OFC was positive in 31 cases, including 10 cases of anaphylaxis with lower respiratory symptoms. Ten of these 31 children (2 of 10 anaphylaxis reactions) had a positive LDC to egg (2), cow's milk (1), peanut (1), cashew (2), hazelnut (1), salmon (1), chickpea (1), and prawn (1). In these children with both a positive LDC and OFC, 5 reacted to the top dose (∼3 g protein) while 3 reacted to the first dose of the OFC. Two children with positive LDCs went on to have a negative diagnostic OFC (one to egg, the other to sardine fish), giving a false-discovery rate (the probability of a false positive if LDC is positive) of 17% (2 of 12). Diagnostic characteristics of the LDC are presented in Table III. There were no significant associations between LDC outcome and age (*P* = .17), skin prick test result (*P* = .053), having atopic dermatitis (*P* = .58), the particular food tested (*P* = .80), or in those with a positive OFC, the eliciting dose (*P* = .88) or the occurrence of anaphylaxis (*P* = .24) (see Table E2 in this article's Online Repository at www.jaci-inpractice.org).

**Table II:** Patient characteristics undergoing LDC/OFC (n = 220)

Characteristic	Value
Age (y), median (range)	6.0 (0.4-18)
Skin prick test (mm), median, (range)	2 (0-10)
• Sensitization ≥2 mm	148 (67)
Atopic eczema	80 (36)
Other documented food allergies	207 (94)
Food being tested at OFC:	
• Tree nuts	66 (30)
○ Hazelnut	21 (10)
○ Almond	14 (6)
○ Cashew	12 (5)
○ Walnut	11 (5)
○ Other	9 (4)
• Peanuts	36 (16)
• Egg	35 (16)
• Fish	23 (10)
• Cow's milk	13 (6)
• Prawn/shrimp	9 (4)
• Other	37 (17)
Previous reaction to food being tested	71 (32)

Values are n (%) unless otherwise indicated.

**Table III:** Diagnostic properties of the LDC in relation to OFC outcome in 198 children with suspected IgE-mediated food allergy

Diagnostic properties of LDC	Value
True negative (no. of patients)	165
True positive (no. of patients)	10
False negative (no. of patients)	21
False positive (no. of patients)	2
Specificity (%, 95% CI)	98% (95.3-99.8)
Sensitivity (%, 95% CI)	32% (17.3-51.5)
Positive predictive value (%, 95% CI)	83% (50.9-97.0)
Negative predictive value (%, 95% CI)	89% (83.0-92.7)
Positive likelihood ratio	26.9
Negative likelihood ratio	0.69
False-negative rate	68%
False-positive rate	1.2%

## Discussion

Our data indicate that LDCs are used by most UK pediatric allergy centers as part of their food challenge protocols, despite a lack of evidence on their diagnostic value. We found wide variation in how LDCs are performed and interpreted by health care professionals, which raises major concerns about the reproducibility and the validity of the test. We subsequently evaluated the diagnostic utility of LDCs in a representative clinical cohort, and demonstrated a relatively poor correlation between the outcome of the LDC and the OFC itself.

In our cohort, a positive LDC was uncommon, even in those cases with a positive OFC. Thus, the sensitivity of LDC is low. Where LDC was positive, this was associated with a low false-positive rate and a high positive likelihood ratio, although we observed 2 cases where LDC was positive but participants went on to tolerate the food being tested at OFC, without reaction. These observations lead us to question the value of LDC in clinical practice, especially given the concerns regarding heterogeneity in test performance and interpretation: a high positive likelihood ratio is unlikely to change posttest probability in patients likely to be clinically allergic (and therefore already have a high pretest probability),^13^ while the false-discovery rate (17% in our cohort) despite the use of objective criteria to assess LDC implies a need to proceed to OFC irrespective of LDC outcome.

Our findings with respect to false-positive LDC are consistent with the observations of Hourihane et al.^6^ Venter et al^14^ also assessed the diagnostic utility of LDC, and found that all 9 of 13 patients with a positive LDC also had a positive OFC; however, 4 children did not proceed to formal food challenge. Both Rance and Dutau^4^ and ourselves observed children who developed systemic symptoms following LDC; thus, the same safety measures should be in place for LDC as for routine OFC. It is therefore difficult to see how incorporating LDC before OFC might be cost-effective, given the low positive rate and the precautions required because of risk of systemic reaction. In addition, the inclusion of LDC prolongs the challenge test unnecessarily. We used objective criteria consistent with the PRACTALL consensus to determine the outcome of OFC, in contrast to previous studies. We therefore believe that our data provide evidence against the use of LDC during OFC and certainly as an alternative to OFC, due to relatively poor diagnostic utility (in part because of a lack of clarity over test interpretation), as well as the potential for systemic reactions.

Although our prospective assessment of LDC was limited by cohort size, our data are representative of children undergoing OFC in routine clinical practice, and our sample size is probably typical of the number of food challenges that may be undertaken by a medium-to large-size allergy service over a 1-year period. For example, a large tertiary allergy service in the United States undertook an average of 230 OFCs per annum, although this cohort had a positive OFC rate of 30%.^15^ The lower rate of positive OFC in our cohort implies a lower risk profile to included cases. Thus, the diagnostic characteristics of LDC generated in this study cannot necessarily be applied elsewhere, and although our cohort was sufficiently powered to assess specificity (and thus rate of false positives), we were underpowered to estimate sensitivity/positive predictive value as indicated by the lower precision of the estimates as seen in the 95% CIs for sensitivity/positive predictive value in Table III. In routine practice, outside the context of desensitization, OFCs are generally performed in patients in whom the degree of sensitization is less than that which predicts 95% likelihood of reaction. However, OFCs are increasingly undertaken in children with a higher likelihood of clinical allergy—for example, before the commencement of oral immunotherapy. In this context, it is possible that the false-positive rate would be slightly lower, although this does not negate the significance of a false-positive LDC as observed in this study. We would not recommend LDC as part of OFC before initiating oral immunotherapy, because in this context one should define an eliciting dose (which then allows response to treatment to be assessed) and not depend on LDC that is clearly associated with a false-positive rate.

Recent research has provided evidence for the earlier introduction of potential food allergens into the infant diet, to reduce the risk of developing food allergy.^16^ Although the associated guidelines do not suggest LDC,^17^ in our experience many health care professionals recommend LDC as an initial step in introducing foods such as peanut butter into the infant diet. We caution against this approach, given the lack of evidence in the literature and the false-discovery rate identified in this study.

In conclusion, current guidelines should counsel against the use of LDC in the diagnosis of IgE-mediated food allergy, because there is no robust evidence that LDC is useful for diagnosis. Finally, units continuing to perform LDC either as part of routine protocols or at parental request should interpret any positive findings with care and preferably proceed to a routine OFC.

## Figures and Tables

**Table E1:** Questions used in this survey and responses obtained

Question	Response options	Responses obtained, n (%)
Q1. Please select your main role as a health professional.	Dietitian	9 (5.7)
	Medical doctor	92 (58.2)
	Nurse/nurse practitioner	51 (32.3)
	Other	6 (3.8)
Q2. A. If you are a dietitian, in what area of dietetics do you work?	General dietetics	0 (0)
	Pediatric dietetics	5 (55.6)
	Specialist allergy dietetics	4 (44.4)
	Other	0 (0)
Q2. B. If you are a doctor, please select which option best describes your medical practice.	Specialist in allergy/immunology	41 (44.6)
	Adult physician (hospital based)	6 (6.6)
	General pediatrics	39 (42.4)
	General practice	4 (4.4)
	Other	2 (2)
Q2.C. If you are a nurse, please select the option which best describes your area of practice.	Specialist allergy nurse	41 (80.4)
	Pediatrics	8 (15.7)
	Adult nursing	2 (3.9)
	Community nurse/nurse in general practice	0 (0)
	Other	0 (0)
Q3. Do you work in	A tertiary specialist allergy clinic	67 (48)
	District general hospital	63 (49.6)
	Primary care facility	3 (2.4)
Q4. For how many years have you been performing food challenges yourself?	Over 10 y	56 (37.1)
	5-10 y	46 (30.5)
	Under 5 y	37 (24.5)
	I am still in training	12 (8)
Q5. The patients I see are	All pediatric patients	100 (86.7)
	Mostly pediatric but some adult patients	7 (4.7)
	About half pediatric and half adult patients	2 (1.3)
	Mostly adult but some pediatric patients	17 (11.3)
	All adult	24 (16)
Q6. How many food challenges does your unit perform every month, on average?	<5 per month	41 (28)
	5-10	32 (21.9)
	10-25	33 (22.6)
	25-50	26 (17.8)
	More than 50	14 (9.6)
Q7. Do you think “lip doses” are useful as a first step in performing a food challenge?	Yes	101 (69.7)
	No	33 (22.8)
	Not sure	11 (7.6)
Q8. Do you (or your unit) ever use lip doses when doing a food challenge?	Yes	117 (80.7)
	Never	28 (19.3)
Q9. How do you perform a lip dose to cow's milk?	Apply a drop to the inner lip	49 (45)
	Apply a drop to the outer lip	48 (44)
	Other	12 (11)
Q10. How do you perform a lip dose to peanut?	Apply a smear of peanut butter to the inner lip	15 (13.8)
	Apply a smear of peanut butter to the outer lip	19 (17.4)
	Rub some peanut on the inner lip	37 (33.9)
	Rub some peanut on the outer lip	29 (29.6)
	Other	9 (8.3)
Q11. How do you perform a lip dose to cashew nut?	Rub some cashew on the inner lip	50 (48.3)
	Rub some cashew on the outer lip	48 (44.4)
	Other	10 (9.3)
Q12. What do you consider to be a positive lip dose (tick all that apply)?	Patient complains of itchy lip or mouth	38 (36.5)
	Redness or erythema at the site of application of dose	48 (46.2)
	Urticaria and/or swelling at the site of application	97 (93.3)
	Urticaria/angioedema on the face, but away from the site of application	77 (74)
	Urticaria on the body	73 (70.2)
Q13-17. If the following symptoms occurred to a lip dose, what would you do?		
Q13. Patient complains of itchy lip or mouth	STOP the challenge, ie, not proceed to an oral dose	18 (17.3)
	CONTINUE with the challenge, ie, proceed to an oral dose	86 (82.7)
Q14. Redness or erythema at the site of application of dose	STOP the challenge, ie, not proceed to an oral dose	32 (30.8)
	CONTINUE with the challenge, ie, proceed to an oral dose	72 (69.2)
Q15. Urticaria and/or swelling at the site of application	STOP the challenge, ie, not proceed to an oral dose	91 (87.5)
	CONTINUE with the challenge, ie, proceed to an oral dose	13 (12.5)
Q16. Urticaria/angioedema on the face, but away from the site of application	STOP the challenge, ie, not proceed to an oral dose	97 (93.3)
	CONTINUE with the challenge, ie, proceed to an oral dose	7 (6.7)
Q17. Urticaria on the body	STOP the challenge, ie, not proceed to an oral dose	97 (93.3)
	CONTINUE with the challenge, ie, proceed to an oral dose	7 (6.7)

**Table E2:** Comparative analysis between children with positive and negative LDCs (N = 198)

Characteristic	LDC result		*P* value
	Positive	Negative	
Age (y), median	3.0	6.0	.17
Atopic dermatitis, n (%)	6 (50)	64 (34)	.58
Skin prick test (mm), median	3	2	.053
Food tried at challenge, n (%)			.80
Tree nuts	3 (25)	58 (31)	
Peanut	1 (8)	32 (17)	
Egg	3 (25)	30 (16)	
Fish	2 (16.7)	18 (10)	
Cow's milk	1 (8)	11 (6)	
Prawn/shrimp	0 (0)	7 (4)	
Other	2 (17)	30 (16)	
Anaphylaxis at OFC (where OFC positive)	2 of 10 (20)	8 of 21 (38)	.24
